# The Right Direction Needed to Develop White-Box Deep Learning in Radiology, Pathology, and Ophthalmology: A Short Review

**DOI:** 10.3389/frobt.2019.00024

**Published:** 2019-04-16

**Authors:** Yoichi Hayashi

**Affiliations:** Department of Computer Science, Meiji University, Kawasaki, Japan

**Keywords:** deep learning, white box, interpretability, transparency, rule extraction, radiology, pathology, black box

## Abstract

The popularity of deep learning (DL) in the machine learning community has been dramatically increasing since 2012. The theoretical foundations of DL are well-rooted in the classical neural network (NN). Rule extraction is not a new concept, but was originally devised for a shallow NN. For about the past 30 years, extensive efforts have been made by many researchers to resolve the “black box” problem of trained shallow NNs using rule extraction technology. A rule extraction technology that is well-balanced between accuracy and interpretability has recently been proposed for shallow NNs as a promising means to address this black box problem. Recently, we have been confronting a “new black box” problem caused by highly complex deep NNs (DNNs) generated by DL. In this paper, we first review four rule extraction approaches to resolve the black box problem of DNNs trained by DL in computer vision. Next, we discuss the fundamental limitations and criticisms of current DL approaches in radiology, pathology, and ophthalmology from the black box point of view. We also review the conversion methods from DNNs to decision trees and point out their limitations. Furthermore, we describe a transparent approach for resolving the black box problem of DNNs trained by a deep belief network. Finally, we provide a brief description to realize the transparency of DNNs generated by a convolutional NN and discuss a practical way to realize the transparency of DL in radiology, pathology, and ophthalmology.

## Introduction

Recently, deep learning (DL) has become an increasingly popular trend in the machine learning community. The theoretical foundations of DL are well-rooted in the classical neural network (NN) literature. Among the DL architectures, convolutional NNs (CNNs) have made the greatest impact in the field of computer vision (Ravì et al., 2017). CNNs, which are composed of convolutional, pooling, and fully connected layers, are feedforward networks in which information flow occurs in one direction only, from input to output. Similar to artificial NNs (ANNs), CNNs are biologically inspired. To attain the desired network output, CNNs utilize learning algorithms to adjust their free parameters (i.e., biases and weights). Backpropagation (BP) is the most common algorithm used for this purpose (Rumelhart et al., 1986).

A new multi-layered NN model proposed by Fukushima (1979), the *neocognitron*, was found to be successful at recognizing simple input patterns regardless of a shift in position or distortions in the shape of the input pattern (Fukushima, 1980). This model laid the foundation for the development of CNNs (Rawat and Wang, 2017). As CNNs were derived from the neocognitron, they have a similar architecture (LeCun et al., 2015).

In 1989, LeCun et al. (1989a,b) proposed the first multi-layered CNNs and successfully applied these large-scale networks to real image classification problems. These initial CNNs were reminiscent of the neocognitron (Fukushima, 1979). CNNs have been applied to visual tasks since the late 1980s. In 1998, CNNs (LeCun et al., 1989a,b) were improved upon and used for individual character classification in a document recognition application. LeCun et al. (1998) introduced the popular Modified National Institute of Standards and Technology (MNIST) dataset (LeCun et al., 1998), which has since been used extensively for a number of computer vision tasks.

However, despite their use in several applications, they remained largely underutilized until about a decade ago, when developments in computing power, improved algorithms, and the advent of large amounts of labeled data contributed to their advancement and brought them to the forefront of a NN renaissance (Rawat and Wang, 2017).

Other plausible architectures for DL include those grounded in compositions of restricted Boltzmann machines (Freund and Haussler, 1991) such as deep belief networks (DBNs; Hinton and Salakhutdinov, 2006), which extend ANNs with many layers as deep NNs (DNNs). Prior to this, it was assumed that DNNs were too hard to train due to issues with gradient descent, and thus, not very popular (Bengio et al., 2006).

In contrast to the NN renaissance, careful attention should be paid to the *hidden* shadow side. Our motivation is to explore the shadow side from the viewpoint of “*white box*” DL. That is, we should clarify the reason why black-box machine learning, such as CNNs, works well for classification tasks in radiology, pathology, and ophthalmology. Therefore, the aim of the present paper is to review the fundamental limitations and criticisms of DL in radiology, pathology, and ophthalmology and in the conversion from DNNs to decision trees (DTs). We demonstrate transparent approaches for resolving the “black box” nature of DBNs and describe future aspects to realize the transparency of DL in radiology, pathology, and ophthalmology.

## Rule Extraction and the “Black Box” Problem

Rule extraction is not a new concept, but was originally raised for a shallow NN by Gallant (1988) and Saito and Nakano (1988) for the medical domain. For about the past 30 years, extensive efforts have been made by many researchers to resolve the “black box” problem of trained NNs using rule extraction technology (Hayashi, 1991, 2016, 2017; Andrews et al., 1995; Craven and Shavlik, 1996; Tickle et al., 1998; Mitra and Hayashi, 2000; Bologna, 2001; Setiono et al., 2008;Tran and Garcez d'Avila, 2016).

Rule extraction (Andrews et al., 1995) is a powerful and increasingly popular method of data mining that provides explanations and interpretable capabilities for models generated by shallow NNs. Extracted rules need to be simple and interpretable by humans, and must be able to discover highly accurate knowledge in the medical and financial domains. Rule extraction technology has also been recognized as a technique that attempts to find a compromise between the two requirements (accuracy and interpretability) by building a simple rule set that mimics how a well-performing complex model (“black box”) makes decisions for users (Fortuny and Martens, 2015). Therefore, high-performance classifier research (Tsai, 2014) seems to maintain a sole focus on predictive accuracy only.

Recently, as a promising means to address the “black box” problem, a rule extraction technology that is well-balanced between accuracy and interpretability was proposed for shallow NNs (Hayashi, 2016). Especially, in rule extraction for medical datasets, there is a trade-off between high classification accuracy and interpretability, such as the number of extracted rules (Hayashi and Yukita, 2016). Very recently, (Hayashi and Oisi, 2018) proposed a high-accuracy priority rule extraction algorithm to enhance both the accuracy and interpretability of extracted rules that is realized by reconciling both of these criterions. In addition, Uehara et al. (2018) reported an actual medical application in hepatology using rule extraction.

## A Renewed Attack of the “Black Box” Problem for Deep Neural Network Architectures

Particularly in cases involving ethics, such as medicine and finance, and in critical applications in which the correctness of a model's prediction must be manually verified, the interpretability of predictive models is important. In fact, the “black box” nature of DL in medicine, especially in radiology, pathology, and ophthalmology, has been severely criticized. Therefore, a “new black box” problem caused by highly complex DNN models generated by DL must be confronted. To resolve this new black box problem, transparency, and interpretability are needed in DNNs.

By contrast, some researchers have investigated the possibility of mapping DTs and random forests into NNs (Biau et al., 2016). For example, Humbird et al. (2018) proposed a deep, jointly-informed NN (DJINN) algorithm map ensemble of DTs trained on the data into a collection of initialized NNs that would then be trained by BP. The authors presented compelling evidence suggesting that DJINNs represented a *robust* “black box” algorithm that could generate accurate NNs for a variety of datasets.

However, at present, various “black box” problems remain for DNNs. By contrast, as machine learning-based predictions become increasingly ubiquitous and affect numerous aspects of our daily lives, the focus of current research has moved beyond model performance (e.g., accuracy) to other factors, such as interpretability and transparency (Yang et al., 2018).

## Approaches to the Transparency of Deep Learning in Computer Vision: The Mnist Case

The MNIST dataset (LeCun et al., 1998) is a difficult problem for rule extraction because the inputs are very low-level abstraction pixels in the images that have to be classified into 10-digit classes. The rules must therefore capture the “hidden” low-level abstraction learned by DL. Such image domains are notoriously difficult for symbolic reasoning (Tran and Garcez d'Avila, 2016).

Zilke et al. (2016) first proposed a new decompositional (Andrews et al., 1995) algorithm called DeepRed (DNN rule extraction via tree induction), which extends the continuous/discrete rule extractor via a DT induction algorithm (Sato and Tsukimoto, 2001). Their approach used C4.5 (Quinlan, 1993) to generate rules using postprocessing that describes rules to produce a rule set that mimics the overall behavior of a given DNN. Although the algorithm was quite useful, it did not work well for the MNIST dataset.

Symbolic rules were initially generated from DBNs by Tran and Garcez d'Avila (2016), who trained a network using the MNIST dataset, obtaining a predictive accuracy of 97.63%, an unknown number of rules with a predictive accuracy of 93.97%, and 784 antecedents per rule, which was equal to the input dimensionality.

As clearly demonstrated by Bologna and Hayashi (2016, 2017), when there are a high number of extracted rules in the practical settings, the entire extracted rule set has no practical significant differences from high-performance classifiers such as a DBN. Bologna and Hayashi (2017) reported 65 extracted rules from the MNIST dataset using discretized interpretable multi-layer perceptron (DIMLP) ensembles (Bologna, 2001), resulting in a predictive accuracy of 97.16% and an average number of 11.1 antecedents per rule.

By contrast, the average number of antecedents per rule obtained by Tran and Garcez d'Avila (2016) was 784. Therefore, from a practical trade-off perspective, there is plenty of room to ensure both interpretability and conciseness, e.g., by decreasing the number of rules extracted and the average number of antecedents per rule.

**Figure 1:**
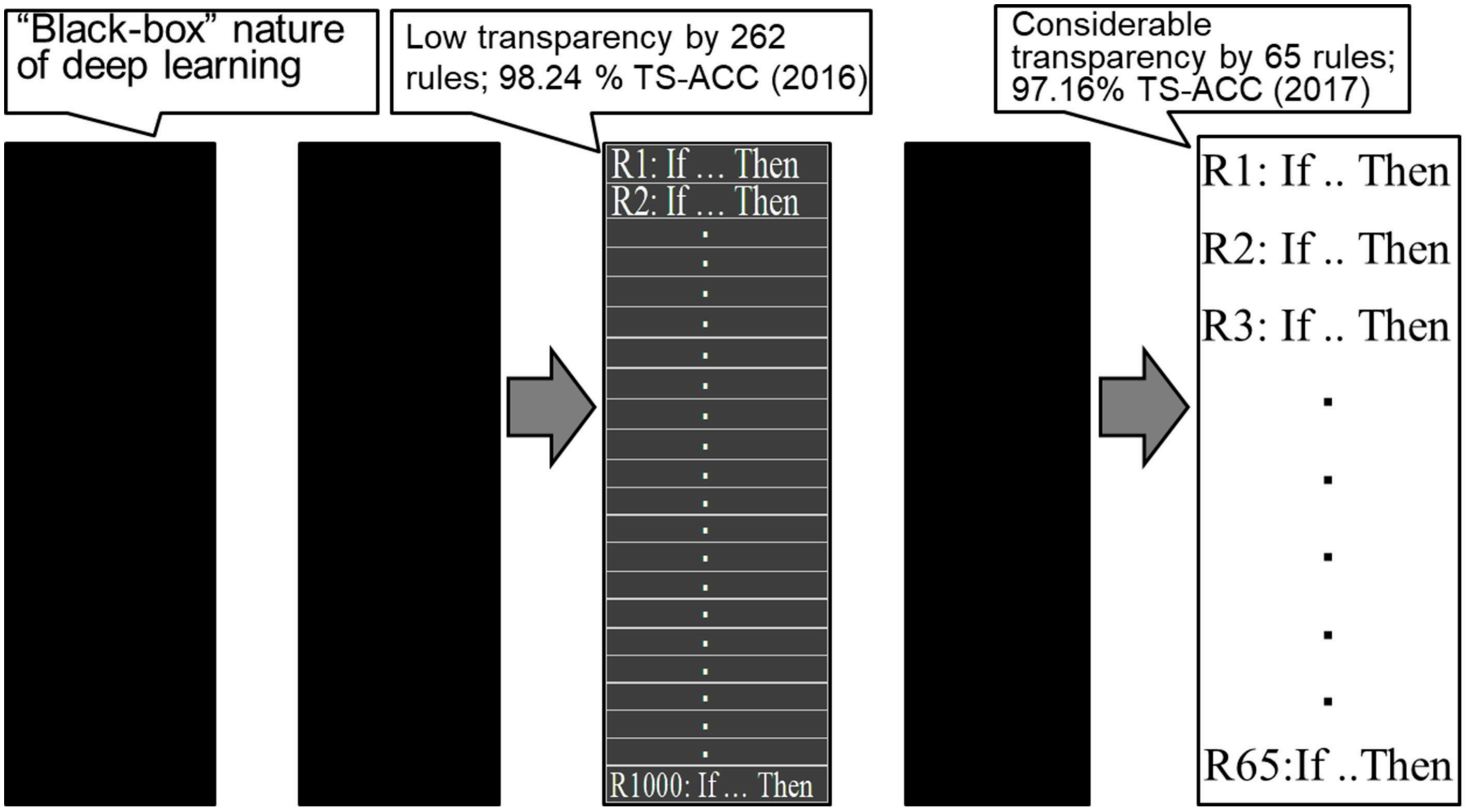
Paradigm shift regarding the transparency of deep learning. TS-ACC, Test accuracy.

This clearly demonstrates a paradigm shift regarding the transparency of DL using rule extraction for the MNIST dataset, as shown in Figure 1. As shown in the figure, starting with the “black box” nature of DL, we first achieved a low level of transparency (Bologna and Hayashi, 2016), followed by a considerable level of transparency (Bologna and Hayashi, 2017).

## Fundamental Limitations and Criticisms of Deep Learning in Radiology, Pathology, and Ophthalmology

### The “Black Box” Nature of Deep Learning in Radiology, Pathology, and Ophthalmology

In contrast to computer vision tasks, DL in radiology, pathology, and ophthalmology still has considerable limitations in terms of its interpretability and transparency. To interpret and apply DL to these medical images effectively, sufficient expertise in computer science is required in the clinical setting. This is because of the “black box” nature of DL, where results are generated with high accuracy with no specific medical-based reason. Hence, the results from DL can be difficult to interpret clinically, which can limit their use in medical decision-making (Vial et al., 2018).

Although some researchers have emphasized the importance of improvements in model performance over interpretability, we feel that improvements in the transparency of DL would promote the widespread adoption of such methods for medical imaging in clinical practice. In addition, context plays a role, as life-and-death decisions made by systems with only marginal improvements in accuracy compared with a human practitioner might warrant greater transparency than those with near-perfect accuracy or lower stakes (Shickel et al., 2018).

Especially in medicine, where accountability is of the utmost importance and can have serious legal consequences, DL is often not sufficient as a prediction system. In regard to outcome prediction, the path toward predictive radiotherapy using DL could still be long. Radiation oncologists should first be capable of understanding predictions that are based on DL algorithms; however, these are still considered “black boxes,” and therefore, their interpretation often remains difficult (Shickel et al., 2018).

We describe four examples of “black box” problems in the following sections. In this paper, the “black box” problem (nature) itself is a major limitation; possible solutions are described in Conversion From Deep Neural Networks to Decision Trees, and Limitations and A Transparent Approach for Resolving the “black box” Nature of Deep Belief Networks.

### Diagnosis of Diabetic Retinopathy

Gulshan et al. (2016) noted the limitations of their system for the detection of diabetic retinopathy. One fundamental limitation inherent to DNNs is that the NN is provided only with the image and associated grade, not with any explicit definitions of the features that would explain the medical diagnosis. The severity of diabetic retinopathy (none, mild, moderate, severe, or proliferative) was graded according to the International Clinical Diabetic Retinopathy scale. Image quality was assessed by graders using the rubric in the “Grading Instructions.” Thus, after the grading, the prediction of a diagnosis of diabetic retinopathy can be formulated as a classification problem; hence, the diagnostic process is a “black box.”

### Histopathological Characterization of Colorectal Polyps

Korbar et al. (2017) noted that the “black box” approach to outcomes was a limitation of DL models in general, and specifically of their system for the histopathological characterization of colorectal polyps to determine the risk of colorectal cancer. Image analysis methods mostly determine the efficacy of the final results, only rarely giving sufficient evidence and details of the factors that contribute to outcomes.

### Breast Density Assessment Using Digital Mammograms

Mohamed et al. (2017) reported that breast density needs to be assessed in the large number of digital mammograms acquired every year in breast cancer screening. However, radiologists may be incapable of reproducing their own assessments, and substantial discrepancies have been observed between different radiologists in regard to assessing a breast as either “scattered density” or “heterogeneously dense.” Although reducing this variation in breast density readings is an urgent clinical need, a better understanding of the aspects regarding how radiologists read images, such as how different views of a mammogram are used, is needed; this issue is closely associated with the “black box” nature of DL.

### Detection of Metastatic Breast Cancer in Sentinel Lymph Node Biopsies

DL in the clinical setting (LYmph Node Assistant: LYNA) has achieved an area under the receiver operating characteristic curve of 99.6% for the detection of metastatic breast cancer in sentinel lymph node biopsies (Golden, 2017), but not without limitations. Although that study tried to unpack the “black box” mechanisms underlying LYNA's predictions by computing the degree to with they were affected by each pixel, LYNA is still unable to compare the current field of view with similar cells in less ambiguous regions of the same slide or case, which can be done by a pathologist (Liu Y. et al., 2018).

## Conversion From Deep Neural Networks to Decision Trees, and Limitations

One approach for understanding DNNs generated by DL is to convert the weights of the fully connected network into a more familiar form. The conversion of network weights of DNNs into DTs, which is basically a series of *if–then* decisions based on criteria used by the network, was proposed by Kontschieder et al. (2015). Although a slight loss in overall accuracy was observed, this approach provided a sense of the information necessary to make decisions.

Subsequently, Zhou and Feng (2017) proposed a method—the multi-Grained Cascade forest (*gc*Forest) method—that can construct a deep forest, which is a deep model based on DTs in which the training process does not rely on BP. Compared with DNNs, the *gc*Forest method has far fewer hyper-parameters, and in their experiments, they could obtain excellent performance across various domains, even when using the same parameter settings. In addition, Yang et al. (2018) proposed a new model at the intersection of DNNs and DTs—a deep neural DT (DNDT), which explores the connections between DNNs and DTs. DNDTs are NNs with a special architecture in which any setting regarding its weights corresponds to a specific DT, thereby making it interpretable. However, because a DNDT is realized by an NN, it inherits several properties that differ from those of conventional DTs. All DNDT parameters are simultaneously optimized using stochastic gradient descent as opposed to a more complex and potentially sub-optimal greedy splitting procedure.

Roy et al. (2018) proposed a network of CNNs, Tree-CNN, that grows hierarchically with the introduction of new classes to the hierarchical structure to avoid catastrophic forgetting (Goodfellow et al., 2013) and leverage the features learned in previous tasks. The branching is based on the similarity of features between the old and new classes. The initial nodes of Tree-CNN assign the input into broad super classes that become more finely classified as they approach the leaves of the network. This type of model allows the convolution layers learned previously to be leveraged and used in the new bigger network. The overall accuracies of Tree-CNN for CIFAR-10 and 100 (Krizhevsky and Hinton, 2009) were shown to be 86.24 and 60.46%, respectively. On the other hand, to the best of our knowledge, these accuracies are considerably lower than state-of-art accuracies, i.e., 95.7% (Li et al., 2018) and 70.8% (Hang and Aono, 2017), respectively. Therefore, Tree-CNN appears to be difficult to apply to the diagnosis of medical images with high accuracy.

Moreover, as Tree-CNN continues to increase in size over time, the implications of this growth on memory requirements, as well as the necessity for storing old training examples, need to be assessed. In this growth, images in Tree-CNN that share common features are closer than those that differ.

Zhang et al. (2019) recently proposed a DT that could explain CNN predictions at the semantic level through the introduction of the following two concepts: *bridging middle-layer features with semantic concepts*, and *bridging middle-layer features with final CNN predictions*. They also developed a unique method for revising CNNs and devised a tight coupling of a CNN and a DT. The proposed DT encodes the decision modes of the CNN as quantitative rationales for each prediction.

Generally, because fine-grained decision modes are close to image-specific rationales, they typically yield lower error prediction rates. However, fine-grained decision modes do not achieve higher classification accuracy because they are designed to mine common decision modes for objects in a certain category while ignoring random/negative images; this process differs from the discriminative learning of classifiers (Zhang et al., 2019).

Therefore, we believe that converting a DNN to DTs cannot be performed in a straightforward manner to realize interpretation of a DNN because large and complex DTs are mathematically equivalent to interpretable DTs, which are not always appropriate for the pre-processing of *if–then* rule expression, and apparently, not interpretable among radiologists, pathologists, and ophthalmologists.

## A Transparent Approach for Resolving the “Black Box” Nature of Deep Belief Networks

As noted by Erhan et al. (2010), in terms of achieving a lower minimum of the empirical cost function, unsupervised pre-training initializes a model to a point in the parameter space that renders the optimization process more effective. The same difficulties are also confronted during the BP learning process. Therefore, in the supervised learning phase, the learning of input information from the feature space by the DBN could initialize the BPNN to well converge an objective function into a near good local optimum, called DBN-NN; this could be the rationale behind the enhancement made possible by a simple idea (Abdel-Zaher and Eldeib, 2016).

The large margin principle (Erhan et al., 2010) can generally be applied to rating category datasets with relatively high numbers of features (attributes) such as biomarkers or radiologists' readings. In fact, very recently, Hayashi (2018) proposed a new method, called DBN Re-RX with J48graft, to extract accurate and interpretable classification rules for DBNs. He applied this method to three rating category datasets (Luo et al., 2017)—the Wisconsin Breast Cancer Dataset^1^, the Mammographic Mass dataset^1^, and the Dermatology dataset^1^—all three of which are small, high-abstraction datasets with prior knowledge. After training these three datasets, he proposed a rule extraction method that could extract accurate and concise rules for DNNs trained by a DBN. These results suggested that the Re-RX family (Hayashi, 2016) could help fill the gap between the very high learning capability of DBNs and the very high interpretability of rule extraction algorithms such as Re-RX with J48graft (Hayashi, 2017). Therefore, a better trade-off between predictive accuracy and interpretability can be achieved in not only rating category datasets, but also image datasets consisting of relatively high-level abstract features.

A comparison of classification using DBN-NN and rule extraction using DBN Re-RX with J48graft for DBNs is shown in Table 1.

**Table 1:** Comparison of classification using DBN-NN and rule extraction using DBN Re-RX with J48graft for DBNs.

	**Method to achieve better initialization**	**Rationale for better initialization**	**Transfer of weights**	**Main component**	**Advantages**	**Limitations**
DBN-NN	DBN	Large margin principle	Full transfer	BP	High classification accuracy	No transparency
DBN Re-RX with J48graft	DBN	Large margin principle	One-to-one mapping	Re-RX with J48graft	Rule extraction (transparency) and classification	Slightly lower classification accuracy

*DBN, Deep belief network; NN, Neural network; Re-RX, Recursive-Rule eXtraction*.

## Future Aspects to Realize the Transparency of DL in Radiology, Pathology, and Ophthalmology

We can extend DBN Re-RX with J48graft (Hayashi, 2018) to “CNN Re-RX” for high-level abstraction datasets using fully connected layer-first CNNs (FCLF-CNNs), in which the fully-connected layers are embedded before the first convolution layer (Liu K. et al., 2018), because the Re-RX family (Hayashi, 2016) uses DTs such as C4.5 (Quinlan, 1993) or J48graft (Hayashi, 2017).

In general, we can extract rules using pedagogical (Andrews et al., 1995) approaches such as C4.5, J48graft, the Re-RX family, Trepan (Craven and Shavlik, 1996), and ALPA (Fortuny and Martens, 2015), regardless of the input and output layers in any type of DL for images with high-level abstraction attributes with prior knowledge. For more details, we will present a concrete method in another paper. However, medical images in radiology, pathology, and ophthalmology are not always provided in a sufficiently high degree of abstraction datasets with prior knowledge. A practical way to avoid this difficulty is to pay attention to the high-level abstraction of attributes associated with medical images.

For example, the digital database for screening mammography (DDSM) (Michael et al., 1998, 2001) consists of mammographic image assessment categories for the breast imaging reporting and data system (BI-RADS) (Obenauer et al., 2005) and the nominal attributes of breast density and patient age. Current approaches using DL are also reliable for various image pre-processing techniques, such as a region of interest selection, segmentation, and feature extraction.

Using data from regularly screened women based on results from a single screening round using digital mammography, Nelson et al. (2016) reported that the false-positive rate was highest among those aged 40–49 years (12.1%), and that the false-negative rates among all women ranged from 0.1 to 0.15%. In two different studies (Moss et al., 1999; Moy et al., 2002), 0.4–3.7% of breast cancers showed false-negative findings on mammography and ultrasound (Chan et al., 2015).

Therefore, we feel that the very high classification accuracy (97.35%) for the DDSM obtained by DL using above pre-processing techniques (Ribli et al., 2018) is often overestimated and somewhat optimistic. In this case, the pre-processing techniques mentioned above make the transparency of DL more difficult.

We can extract important rules from attributes associated with medical images using the Re-RX family. However, the classification accuracy using extracted rules is slightly lower than that using whole images trained by a CNN, so we should recognize that to establish accountability, one of the most important issues in medical imaging is to explain the classification results clearly.

## Conclusion

We have provided a review regarding the right direction needed to develop “white box” DL in radiology, pathology, and ophthalmology. We believe that the most important point to realize the transparency of DL in radiology, pathology, and ophthalmology is not that driven features rely on filter responses solicited from a large amount of training data, which suffer from a lack of direct human interpretability; rather, we should utilize the high-level abstraction of attributes associated with medical images with prior knowledge graded and/or rated by radiologists, pathologists, and ophthalmologists.

It should be noted that theoretically, the DT provides only an approximate explanation of CNN predictions, as opposed to an accurate reconstruction of CNN representation details. In radiology, pathology, and ophthalmology, a conversion method of CNN to DTs should be developed with greater preservation of accuracy and interpretability (more concise and less complex). The key points shared by DBN-NN, DBN Re-RX with J48graft, and CNN-Re-RX involve capturing high-level abstraction of unstructured data such as images.

Very recently, Hosaka (2019) attempted to apply a CNN to the prediction of corporate bankruptcy, which in most cases, is treated as a two-class classification problem. This idea may be promising to rule extraction for time series datasets via CNN representation. However, middle-level abstraction data generated from images enables wider classes of transparency of CNNs, so the question of how to generate middle-level abstraction data for images remains open.

High-dimension *fully connected* layers can easily lead to slow convergence and a risk of overfitting (Srivastava et al., 2014) during the training stage. We hope that CNNs can maintain their considerably high performance even if the feature dimension is low; this would show that the small number of hidden units are capable of training powerful discriminative representations (Xu et al., 2019). In other words, classification accuracies will be saturated with unexpectedly small dimensions of features in image datasets. When these characteristics are utilized, much smaller DTs with approximately the same level of accuracy as the highest accuracy using the conversion of CNNs can be generated; this compactness of DTs would be helpful for the transparency of CNNs.

## Author Contributions

YH researched the bibliography, wrote the first draft, and reviewed the final manuscript to be submitted for publication.

### Conflict of Interest Statement

The author declares that the research was conducted in the absence of any commercial or financial relationships that could be construed as a potential conflict of interest.

## Footnotes

^1^Repository. (2015). *University of California Irvine Learning Repository*, http://archive/ics.uci/edu/
